# Sensitive Electrochemical Detection of Phosphorylated-Tau Threonine 231 in Human Serum Using Interdigitated Wave-Shaped Electrode

**DOI:** 10.3390/biomedicines10010010

**Published:** 2021-12-22

**Authors:** Hien T. Ngoc Le, Sungbo Cho

**Affiliations:** 1 Department of Electronic Engineering, Gachon University, Seongnam-si 13120, Korea; ltnh1809@gachon.ac.kr; 2 Department of Health Sciences and Technology, Gachon Advanced Institute for Health Sciences and Technology (GAIHST), Gachon University, Incheon 21999, Korea

**Keywords:** phosphorylated-tau threonine 231, Alzheimer’s disease, electrochemical biosensor, micro-interdigitated electrode, electrochemical impedance spectroscopy, binding affinity

## Abstract

The development of an electrochemical biosensor for the detection of phosphorylated-tau threonine 231 (p-tau231), a biomarker of Alzheimer’s disease (AD), has yet to be achieved. Therefore, in this study, we developed a simple, small size, cheap, and sensitive electrochemical biosensor based on an interdigitated wave-shaped electrode via an activated self-assembled monolayer to preserve a specific anti–p-tau231 antibody (IWE/SAM/EDC-NHS/anti–p-tau231). Detection of p-tau231 in human serum (HS) using the biosensor was undertaken using electrochemical impedance spectroscopy (EIS). The change in charge-transfer resistance (R_ct_) in the EIS analysis of the biosensor indicated the detection of p-tau231 in HS within a wide linear range of detection (10^−4^–10^1^ ng mL^−1^), and a low limit of detection (140 pg mL^−1^). This lower limit is less than the detection level of p-tau231 in cerebrospinal fluid (CSF) (700 pg mL^−1^) of AD patients and the level of CSF p-tau231 of patients with mild cognitive impairment (501 pg mL^−1^), demonstrating the possibility of using the biosensor in detection of p-tau231 at early stage AD. A high binding affinity and low dissociation constant (K_d_) between anti–p-tau231 and p-tau231 in HS was demonstrated by using a biosensor and K_d_ was 7.6 pM, demonstrating the high specific detection of p-tau231 by the biosensor. The good selectivity of the biosensor for the detection of p-tau231 with differential analytes was also examined in this study.

## 1. Introduction

Alzheimer’s disease (AD) is a continuing brain disorder and is a typical disease of dementia. One of the main pathological proteins of AD is abnormally phosphorylated tau (p-tau) protein, which aggregates in the brain as neurofibrillary tangles, resulting in memory degeneration and dementia syndrome [1]. A number of phosphorylated sites of tau have been identified and used as biomarkers for immunoassays, and cerebrospinal fluid (CSF) assays include p-tau at threonine 231 (p-tau231), threonine 181 (p-tau181), and threonine 217 (p-tau217) [1,2]. Recent reports of immunoassays specify the sensitive detection of differential p-tau231, 181, and 217 proteins in the CSF of patients with AD, revealing reliable p-tau biomarkers for AD diagnosis [1,3]. In particular, a recent study of detection of p-tau231 in the CSF of patients with AD reported a high sensitivity of 85% and a specificity of 97% in separating AD from dementia with Lewy bodies and vascular dementia and indicated the appearance of p-tau231 in the early stages of pathology in patients with AD compared to other p-tau [4], reflecting the beneficial features of p-tau231 as a new biomarker for the early onset of AD pathology. CSF and positron emission tomography (PET) methods were used to determine the concentrations of p-tau and the visualization of p-tau aggregates in the clinic, respectively [5,6]. However, the PET technique is intricate, expensive, and has poor spatial resolution and artifacts of movements [6]. The analysis of CSF is an invasive method that requires the use of a lumbar puncture, which resulted in back pain [5]. Blood (serum or plasma) p-tau231 could be considered as a useful biomarker in the identification pathology of AD owing to its easy sample collection and analysis. However, its utility as a blood serum biomarker is unknown. Hence, the detection of p-tau231 biomarker in serum by a convenient and cost-effective method is warranted in the field of AD diagnosis.

In the field of visible macroscopic optical signals, liquid crystals (LCs) are materials in a state between the isotropic liquid and the crystalline state with stimuli-responsive and optically anisotropic properties; therefore, LCs have been used in developing LC-based biosensors for biomolecule detection and protein–protein binding events. An electrical field is applied to adjust the ordering of LCs between two electrodes to manipulate the light passing through the LCs and, thus, the optical appearance [7,8,9,10]. In the field of electrochemical detection, the advantages and importance of electrochemical biosensors for the determination of acceptable concentrations of biomolecules, drugs, and biomarkers have been reported recently because of their reliability, rapid response, high selectivity, and low cost [11,12,13,14,15,16,17,18,19,20,21]. Micro-interdigitated electrodes have been developed and applied as electrochemical biosensors to improve the detection of biomarkers because of their requirement of a small sample volume compatible with a small sensing area and the interdigitating of the electrode [22,23]. However, sensing performance can be decreased in the regular rectangular interdigitated electrode owing to the extensive distribution of electric fields at the edge of the rectangular electrode. The applied electric field is also one of the influencing factors that have been mentioned in LC-based sensors. Therefore, in this study, we designed and fabricated an interdigitated wave-shaped electrode (IWE) with an electrode finger of 7 μm to avoid the effect of a concentrated electric field at the edge, to eliminate the Warburg diffusion impedance, and to enhance the homogeneity of the electrode sensing area, resulting in the improvement of the capabilities of the electrochemical biosensor in biomarker sensing.

To date, an electrochemical biosensor for the detection of p-tau231 protein has not been developed. In this study, an electrochemical biosensor for the detection of p-tau231 protein was developed using an IWE. The IWE was functionalized by an activated self-assembled monolayer (IWE/SAM/EDC-NHS), and then a specific anti–p-tau231 antibody was immobilized on the functionalized electrode to create the biosensor IWE/SAM/EDC-NHS/anti–p-tau231 for the specific detection of p-tau231 protein. Electrochemical impedance spectroscopy (EIS) was used to measure the change in charge-transfer resistance (R_ct_) of the biosensor during the detection of p-tau231. Detection of p-tau231 in human serum (HS) using the developed biosensor revealed a wide linear range of detection (LRD) from 10^−4^ to 10^1^ ng mL^−1^ and a low limit of detection (LOD) of 140 pg mL^−1^, as compared to the average detection level of CSF p-tau231 (700 pg mL^−1^) in patients with AD [4,24], and the level of CSF p-tau231 of patients with mild cognitive impairment (501 pg mL^−1^) [24], demonstrating the capability of the biosensor in the early detection of p-tau231 in the HS. Furthermore, the binding affinity between anti–p-tau231 and p-tau231 was explored by calculating the dissociation constant (K_d_), yielding the low value of K_d_ of 7.6 pM in HS, which is indicative of the high specific binding between anti–p-tau231 and p-tau231 and confirmed the high specificity toward the detection of p-tau231 using the biosensor. The selectivity of the developed biosensor for the detection of p-tau231 in the presence of various analytes was also examined in this study.

## 2. Materials and Methods

### 2.1. Materials

6-mercaptohexanoic acid (90%), N-ethyl-N-(3-dimethylaminopropyl)-carbodiimide hydrochloride (EDC, crystalline), N-hydroxysuccinimide (NHS, 98.0%), human serum (HS, from male AB clotted whole blood, USA origin, sterile-filtered), and potassium ferrocyanide/ferricyanide (K[Fe(CN)_6_]^3^^−/4^^−^) were purchased from Sigma-Aldrich (Seoul, Korea). Potassium chloride (KCl, extra pure) was purchased from Daejung Chemicals & Metals Co., Ltd. (Siheung-si, Korea). Recombinant anti–tau phospho T231 (anti–p-tau231) antibody EPR2488 and phospho-tau231 (p-tau231) blocking protein were purchased from Abcam (Cambridge, UK). Phosphate-buffered saline (PBS, pH 7.4) and bovine serum albumin (BSA, 0.5% in 1X PBS, pH 7.4) were obtained from Tech and Innovation (Shuncheon-si, Korea). De-ionized (DI) water (18.2 MΩ·cm) was obtained from a Milli-Q system (Seoul, Korea).

### 2.2. Manufacturing of the IWE

**Figure 1:**
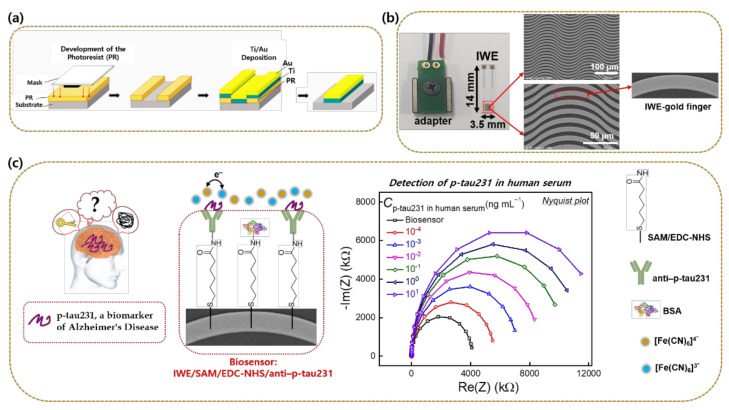
(**a**) Schematic of the fabrication of IWE based on the lift-off process; (**b**) photograph of the homemade adapter, the IWE, and scanning electron microscope images of IWE at different magnifications; (**c**) construction of the biosensor IWE/SAM/EDC-NHS/anti–p-tau231 for the electrochemical detection of p-tau231 via EIS recording.

The IWE was fabricated on a glass slide substrate (14 × 3.5 mm). An electron beam evaporator was used to deposit an adhesive titanium layer with a thickness of 25 nm and a gold electrode layer with a thickness of 50 nm. A lift-off process was then applied to create the paired electrode finger with 7 μm spacing and width for the working and reference electrodes, respectively. The IWE was placed in a homemade adapter and connected to a Bio-Logic sp-200 (Seyssinet-Pariset, France) electrochemical workstation for impedance measurement in a two-electrode configuration. A photograph and scanning electron microscope images of the IWE are shown in Figure 1.

### 2.3. Construction of the Biosensor IWE/SAM/EDC-NHS/anti–p-tau231

The bare IWE was cleaned with ethanol, deionized water, and dried under flowing nitrogen gas to remove contaminants from the IWE’s surface. Next, the IWE was incubated in a 6-mercaptohexanoic acid solution (100 mM) for 24 h to create a self-assembled monolayer (SAM) on the gold electrode surface via sulfur–gold bonding. The IWE with the modification of SAM was subsequently dipped in a solution of EDC (75 mM)/NHS (5 mM) for 1 h to activate the carboxyl group of SAM on the electrode surface ready for anti–p-tau231 antibody binding. After activating the SAM-modified electrode by EDC/NHS, 10 μL of anti–p-tau231 antibody (40 μg mL^−1^) was dropped and immobilized on the electrode surface through interaction of the amino group between the antibody molecule and the activated electrode surface, and the immobilization process was conducted in a wet chamber for 1 h in order to avoid evaporation. Blockage of the nonspecific adsorption on the electrode surface was then attained using BSA (0.5% in 1X PBS, pH 7.4), thereby producing the finished biosensor IWE/SAM/EDC-NHS/anti–p-tau231.

### 2.4. Investigation of the Modification of SAM on the IWE Surface

Energy-dispersive X-ray spectroscopy (EDS, S-4700, HITACHI, Tokyo, Japan) was used to characterize the elemental percentages of samples. Atomic force microscopy (AFM, NX-10, Park Systems, Seoul, Korea) using the non-contact head mode with scan rate of 1 Hz was used to characterize 3D topography and roughness of the electrode surface.

### 2.5. EIS Measurement

A Bio-Logic sp-200 (France) electrochemical workstation was used to measure the EIS of the bare electrode and the modified electrode in a two-electrode configuration in 1 mM K[Fe(CN)_6_]^3^^−/4^^−^ containing 0.1 M KCl. A range of frequencies from 1 MHz to 100 mHz and a sinus amplitude of 50 mV were applied to perform EIS. Various concentrations of p-tau231 protein, from 10^−4^ to 10^1^ ng mL^−1^, were prepared in PBS (pH 7.4) and HS (diluted with PBS at a ratio of 1:100 to avoid the matrix effect of HS). The prepared p-tau231 proteins in PBS or HS (10^−4^–10^1^ ng mL^−1^) were then incubated with the biosensor for 20 min at room temperature (25 °C), and EIS measurements were conducted and recorded. Z-Fit software (EC-Lab, Bio-Logic, sp-200, France) was used to fit EIS data via Randle’s equivalent circuit model.

### 2.6. Cyclic Voltammetry (CV) Measurements

The development of the biosensor at each stage was confirmed by CV measurements using a Bio-Logic sp-200 electrochemical workstation in a three-electrode configuration, including the modified electrode as the working electrode, Ag/AgCl as the reference electrode, and a Pt coil as the counter electrode in 1 mM K[Fe(CN)_6_]^3^^−/4^^−^ containing 0.1 M KCl.

## 3. Results

### 3.1. Characterization the Modification of SAM on the IWE

**Figure 2:**
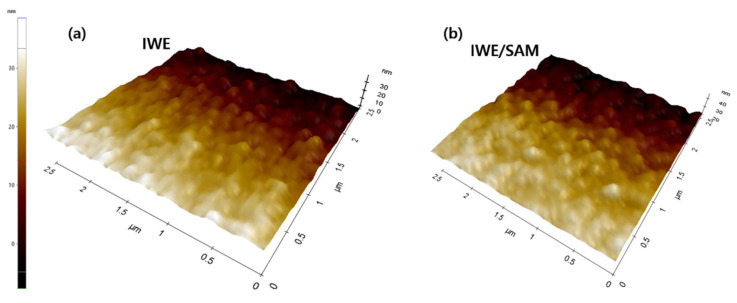
AFM results of (**a**) bare IWE; (**b**) IWE with the modification of SAM on the surface (IWE/SAM).

**Figure 3:**
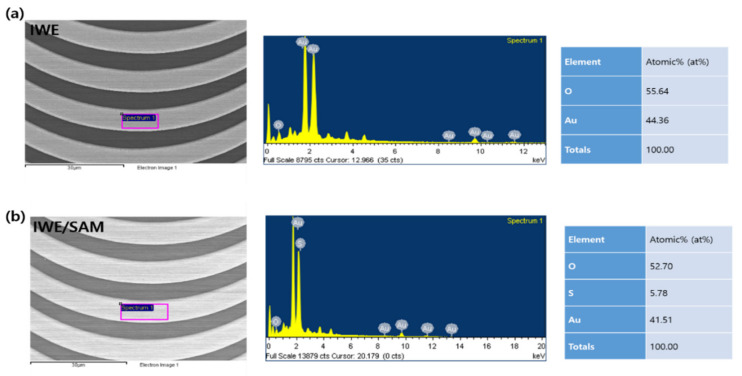
EDS results of (**a**) bare IWE; (**b**) IWE with the modification of SAM on the surface (IWE/SAM).

The formation of SAM on the gold surface of IWE via gold–sulfur bonding at the terminal sulfur group [25] is an important step in the biosensor development process to attach the antibody at the end of the carbodiimide group of SAM activated by the EDC/NHS [26]. In order to confirm the formation of SAM on IWE, AFM was used to characterize 3D topography and the roughness of the bare electrode and the electrode modified by SAM (Figure 2). After SAM modification (Figure 2b), the electrode surface became smooth, with a roughness of 7.1 nm compared to a roughness of 8.4 nm of the gold surface of bare IWE (Figure 2a), demonstrating the formation of SAM on IWE. EDS measurement was also used to confirm the elemental atomic percentage of the modification of SAM on the electrode’s surface. The EDS results are shown in Figure 3, where the sulfur (S) element appeared with an atomic percentage of 5.78 (at%) after SAM modification (Figure 3b), indicating the successful formation of SAM on the gold surface of the IWE through the strong bonding of gold–sulfur [25].

### 3.2. Electrochemical Characterization of the Biosensor (IWE/SAM/EDC-NHS/anti–p-tau231)

**Figure 4:**
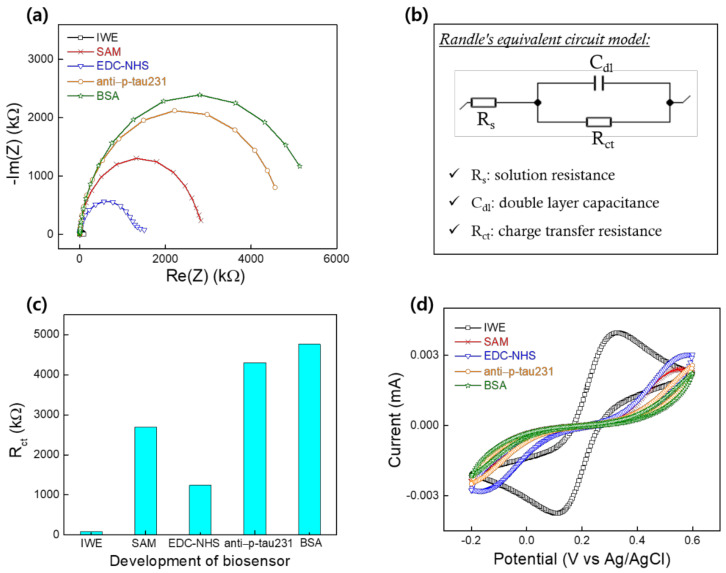
(**a**) Nyquist plots of the biosensor development at each step of IWE, SAM, EDC-NHS, anti–p-tau231, BSA in 1 mM [Fe(CN)_6_]^3^^−/4^^−^ containing 0.1 M KCl; (**b**) Randle’s equivalent circuit model; (**c**) the fitted value R_ct_ from Nyquist plots at each step of the biosensor development; (**d**) CV results at each step of the biosensor development in 1 mM [Fe(CN)_6_]^3^^−/4^^−^ containing 0.1 M KCl.

The development of the biosensor at each step of the modification was illustrated using EIS and CV (Figure 4). EIS was used to explore the change of the interfacial electrode surface through Nyquist plots with imaginary impedance (–Im(Z)) plotted with real impedance (Re(Z)) in 1 mM [Fe(CN)_6_]^3^^−/4^^−^ containing 0.1 M KCl. Figure 4a shows Nyquist plots of the bare IWE and the modification of ingredients to the electrode surface at different steps of the development of the biosensor IWE/SAM/EDC-NHS/anti–p-tau231. The semicircle diameter of the Nyquist plot represents interfacial charge-transfer resistance (R_ct_) in the Randle’s equivalent circuit model. Randle’s equivalent circuit model (Figure 4b) contains solution resistance (R_s_) and double layer capacitance (C_dl_), and R_ct_ was used to fit the Nyquist plot data. The obtained fitting values of R_ct_ of the bare IWE and the modified electrode at each step of SAM, EDC-NHS, anti–p-tau231, and BSA, respectively, are shown in Figure 4c. The value of R_ct_ increased remarkably after the immobilization of SAM owing to the non-conductivity of the long chain of carbon and the COO^–^ group of SAM, which inhibited the transfer of the negatively charged [Fe(CN)_6_]^3^^−/4^^−^ of the redox probe. The CV result of the SAM immobilization (Figure 4d) demonstrated a large decrease in current intensity compared to the CV of bare IWE, confirming the formation of a non-conductive SAM on the surface of the electrode. R_ct_ declined after activating the COO^−^ group of the SAM by the EDC-NHS, demonstrating the complete activation of SAM on the electrode’s surface with the formation of the succinimide ester, which facilitates electron transfer at the electrode interface, corresponding to the increase in current intensity of CV at the EDC-NHS step (Figure 4d). In the next step, with the immobilization of the anti–p-tau231 antibody on the activated electrode surface, R_ct_ increased, which was attributed to the immobilization of an insulating antibody layer on the electrode surface, resulting in a decrease in the corresponding CV (Figure 4d). R_ct_ continuously increased after the deposition of the blocking surface agent BSA, indicating an increase in surface resistance owing to the adsorption of a particular ingredient on the electrode surface, corresponding to the decrease in current intensity in CV at this step (Figure 4d). EIS and CV results confirmed the successful development of the biosensor.

### 3.3. Detection of p-tau231 in PBS and HS using the Biosensor

**Figure 5:**
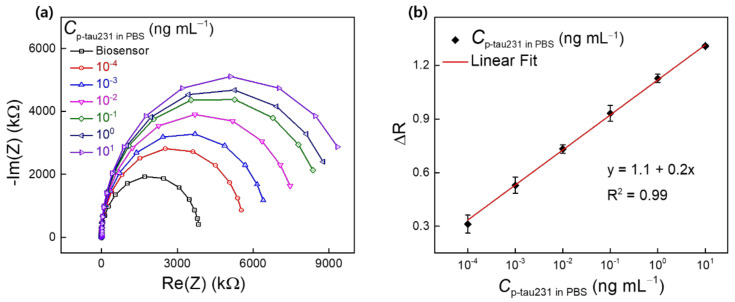
(**a**) Nyquist plots of the detection of p-tau231 in PBS (10^−4^–10^1^ ng mL^−1^) using biosensor IWE/SAM/EDC-NHS/anti–p-tau231; (**b**) calibration curve for the detection of p-tau231 in PBS (10^−4^–10^1^ ng mL^−1^) estimated from the normalization (ΔR) of R_ct_ value in Nyquist plots. Symbols and bars represent the average and standard deviation of the data (*n* = 3).

The procedure for the detection of p-tau231 in PBS and HS using the biosensor IWE/SAM/EDC-NHS/anti–p-tau231 was described in Section 2.5. Figure 5 shows the Nyquist plot of the biosensor after incubation with different concentrations of p-tau231 (10^−4^–10^1^ ng mL^−1^) in PBS. The semicircle diameter or R_ct_ of the biosensor increased gradually after incubation with p-tau231 from low to high concentrations (Figure 5a), indicating the utility of R_ct_ as a parameter to evaluate the detection of p-tau231. The increase in R_ct_ was due to the specific binding of anti–p-tau231 and p-tau231, which blocked electron transfer between the [Fe(CN)_6_]^3^^−/4^^−^ redox probe and the surface of the biosensor, demonstrating the detection of p-tau231 in PBS by the biosensor. Figure 5b shows the calibration curve for the detection of p-tau231 across a range of concentrations from 10^−4^ to 10^1^ ng mL^−1^ in PBS. This was generated by the normalization of the fitted value R_ct_ from Nyquist plots (Figure 5a) via Randle’s equivalent circuit model. Normalization was estimated as ΔR = (R_T_–R_0_)/R_0_, where R_0_ is the R_ct_ of the biosensor, and R_T_ is the R_ct_ of the biosensor after incubation with p-tau231 (10^−4^–10^1^ ng mL^−1^) in PBS. A linear regression equation (y = 1.1 + 0.2x), defined the linear range of detection (LRD) of 10^−4^–10^1^ ng mL^−1^, and the lower limit of detection (LOD) of 0.06 ng mL^−1^ (60 pg mL^−1^) were obtained from the calibration curve for the detection of p-tau231 in PBS. LOD was calculated as (3S/b), where S is the standard deviation and b is the slope of the calibration curve [27].

**Figure 6:**
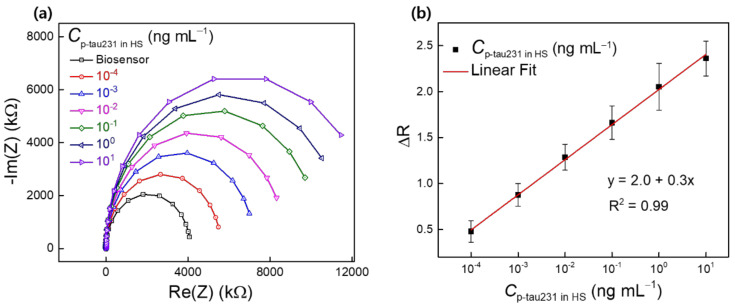
(**a**) Nyquist plots of the detection of p-tau231 in HS (10^−4^–10^1^ ng mL^−1^) using the biosensor IWE/SAM/EDC-NHS/anti–p-tau231; (**b**) calibration curve for the detection of p-tau231 in HS (10^−4^–10^1^ ng mL^−1^) estimated from the normalization (ΔR) of R_ct_ value in Nyquist plots. Symbols and bars represent the average and standard deviation of the data (*n* = 3).

In order to characterize the capability of the biosensor for the detection of p-tau231 in practical applications, the biosensor was used to detect p-tau231 in HS. The measurement process was described in Section 2.5. Figure 6a shows the Nyquist plots for the detection of differential concentrations of p-tau231 in HS (10^−4^–10^1^ ng mL^−1^) using the biosensor. The biosensor exhibited an enhancement of R_ct_ after incubation with p-tau231 in HS, and enhancement gradually increased from 10^−4^ to 10^1^ ng mL^−1^ of p-tau231 concentrations and similar to the trend of detection p-tau231 in PBS, demonstrating the capability of detection p-tau231 in HS. Figure 6b shows the calibration curve for the detection of p-tau231 in HS, estimated from the normalization (ΔR) of the R_ct_ in Nyquist plots and is equal to ΔR = (R_T_–R_0_)/R_0_, where R_0_ is the R_ct_ of the biosensor, and R_T_ is the R_ct_ of the biosensor after incubation with p-tau231 in HS (10^−4^–10^1^ ng mL^−1^). A linear regression equation (y = 2.0 + 0.3x), an LRD from 10^−4^ to 10^1^ ng mL^−1^, and an LOD of 0.14 ng mL^−1^ (140 pg mL^−1^) for the detection of p-tau231 in HS was determined from the calibration curve. Compared to the average detection level of CSF p-tau231 (700 pg mL^−1^) in patients with AD and the detectable concentration of CSF p-tau231 in patients with mild cognitive impairment (501 pg mL^−1^) [4,24], the biosensor exhibited a low LOD of 140 pg mL^−1^ and 60 pg mL^−1^ for p-tau231 in HS and PBS, respectively, demonstrating the capability of the biosensor for the early detection of p-tau231. To date, the study of an electrochemical biosensor for the detection of p-tau231 has not been reported; this is the first study of an electrochemical biosensor for the sensitive detection of p-tau231 in HS and PBS using an IWE.

### 3.4. Binding Affinity, Dissociation Constant (K_d_), and Selectivity and Stability of the Biosensor

**Figure 7:**
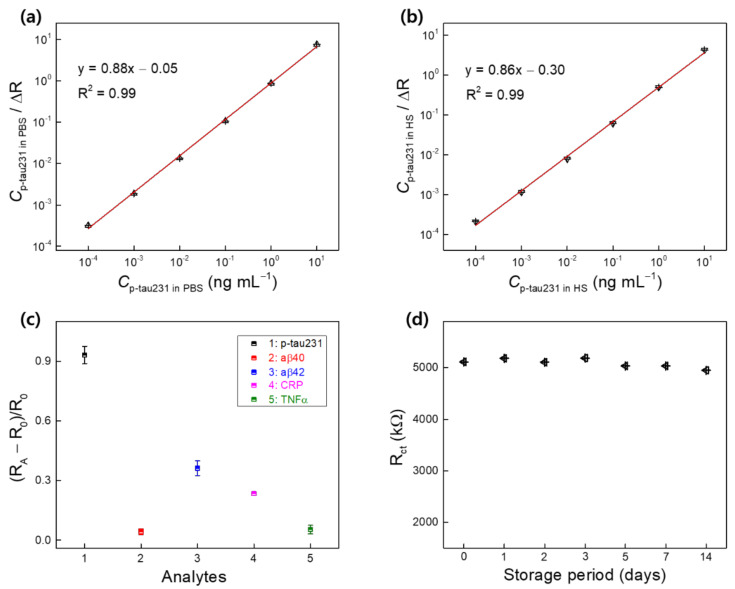
Two linear regressions of C_p-tau231_ versus C_p-tau231_/ΔR in PBS (**a**) and HS (**b**) were plotted to determine the dissociation constant (K_d_); (**c**) selectivity of the biosensor IWE/SAM/EDC-NHS/anti–p-tau231 for the detection of p-tau231 in the attendance of various analytes; (**d**) EIS results of the biosensor were represented by R_ct_ after the storage period.

The Langmuir adsorption model-based approach [28] was used to determine the dissociation constant (K_d_) of the binding between anti–p-tau231 antibody EPR2488 and p-tau231 in this study. K_d_ between anti–p-tau231 and p-tau231 is small, corresponding to a high binding affinity. In our previous study, we estimated K_d_ between antibody and antigen by using the Langmuir adsorption model-based approach [12,29]. Therefore, in this study, a linearized form of the Langmuir isotherm equation, using the response of the biosensor to p-tau231, can be expressed as follows:

C_p-tau231_/ΔR = C_p-tau231_/ΔR_max_ + K_d_/ΔR_max_ (1)

where C_p-tau231_ is the concentration of p-tau231 (ng mL^−1^) in PBS and HS; ΔR is the response normalization to p-tau231 of the biosensor, as indicated in Section 3.3; and ΔR_max_ is the normalization of the maximum response of the biosensor to p-tau231. Using this equation, two linear regressions of C_p-tau231_ versus C_p-tau231_/ΔR in PBS and HS were determined (Figure 7a,b). K_d_ was obtained by dividing the y-intercept by the slope of the linear regression [12,30]. The y-intercept and slope of the two regressions in PBS and HS were 0.05 and 0.88, and 0.30 and 0.86, respectively. Therefore, K_d_ of the binding between anti–p-tau231 antibody and p-tau231 in PBS and HS were 0.056 and 0.35 ng mL^−1^, respectively. The molecular weight of anti–p-tau231 antibody EPR2488 is 46 kDa. Consequently, K_d_ could be converted to 1.2 and 7.6 pM in PBS and HS, respectively. The K_d_ obtained in this study is smaller than estimates from previous studies [31,32,33] and reflects the high binding affinity between anti–p-tau231 antibody EPR2488 and p-tau231 and the high specificity of the recognition of p-tau231. This reconfirms the ability of the biosensor IWE/SAM/EDC-NHS/anti–p-tau231 to detect p-tau231 in PBS and HS.

Figure 7c shows the response (R_A_–R_0_)/R_0_ of the biosensor to various analytes, including p-tau231 (10^−3^ ng mL^−1^), amyloid-β 40 (aβ40, 10 ng mL^−1^), amyloid-β 42 (aβ42, 10 ng mL^−1^), C-reactive protein (CRP, 10 ng mL^−1^), and tumor necrosis factor-alpha (TNFα, 500 pg mL^−1^), where R_0_ is the R_ct_ of the biosensor and R_A_ is the R_ct_ of the biosensor after incubation with different analytes. The response of the biosensor to p-tau231 changed and increased significantly compared to other analytes, indicating specific binding between anti–p-tau231 and p-tau231, confirming the selectivity of the biosensor for the detection of p-tau231. The EIS of the biosensor was measured after 1, 2, 3, 5, 7, and 14 days of storage in the refrigerator at 4 °C, and R_ct_ of the biosensor exhibited minimal change after storage (Figure 7d), indicating the stability of the biosensor.

## 4. Conclusions

Detection of p-tau231 protein, a biomarker for the early onset of AD, is essential for pathological diagnosis. However, to date, the development of an electrochemical biosensor for the detection of p-tau231 has not been realized. Therefore, in this study, a simple, cheap, small, and sensitive electrochemical biosensor was developed on an interdigitated wave-shaped electrode via an activated and self-assembled monolayer to immobilize a specific anti–p-tau231 antibody for the detection of p-tau231. The biosensor exhibited a highly sensitive detection of p-tau231 in HS, with a wide LRD ranging from 10^−4^ to 10^1^ ng mL^−1^, and a low LOD of 140 pg mL^−1^, compared to the detection level of CSF p-tau231 (700 pg mL^−1^) in patients with AD and the detectable concentration of CSF p-tau231 of patients in mild cognitive impairment (501 pg mL^−1^), demonstrating the utility of the biosensor in the early detection of p-tau231 and the diagnosis of AD at the onset stage. The biosensor exhibited a high binding affinity between anti–p-tau231 antibody and p-tau231 protein, with a low dissociation constant K_d_ in HS (7.6 pM), confirming the high specificity toward p-tau231. The biosensor also exhibited good selectivity for the detection of p-tau231 in the presence of various interfering analytes.

## Data Availability

Not applicable.
